# Sex-dependent aortic valve pathology in patients with rheumatic heart disease

**DOI:** 10.1371/journal.pone.0180230

**Published:** 2017-06-29

**Authors:** Feng Xiao, Rui Zheng, Di Yang, Kejiang Cao, Shijiang Zhang, Bingruo Wu, Yongfeng Shao, Bin Zhou

**Affiliations:** 1 Department of Cardiology, The First Affiliated Hospital of Nanjing Medical University, Nanjing, Jiangsu, China; 2 Departments of Genetics, Pediatrics and Medicine (Cardiology), The Wilf Family Cardiovascular Research Institute, Albert Einstein College of Medicine, Bronx, New York, United States of America; 3 Department of Cardiovascular Surgery, The First Affiliated Hospital of Nanjing Medical University, Nanjing, Jiangsu, China; Cincinnati Children's Hospital Medical Center, UNITED STATES

## Abstract

**Background:**

Rheumatic heart disease is an autoimmune disease caused by group A streptococci infection and frequently affects the aortic valve. Sex differences are common in the disease progression, treatment, and outcome. However, little is known about the sex differences in the pathology of aortic valves in rheumatic heart disease.

**Design:**

We studied the end-stage calcific aortic valves from male versus female patients to reveal the sex-dependent pathology differences and molecular changes associated with requiring valve replacement.

**Methods:**

Aortic valves from 39 patients with rheumatic heart disease (19 males and 20 females) were collected at the time of aortic valve replacement for comparative pathology, immunohistochemistry, and gene expression analyses. Clinical characteristics were also analyzed and compared between the two groups.

**Results:**

Aortic valves from female patients exhibited increased expression of collagens, infiltration of monocytes/macrophages and neovascularization. Aortic valves from female patients also had increased expression of inflammatory genes involved in the NFKB pathway (phosphorylated NFKB p65 subunit, IL8, and NOS3) and Th1 cytokine genes (IFNA and IL12B). The severe valve pathology in female patients was correlated with a higher serum level of anti-streptolysin O antibodies.

**Conclusion:**

Inflammation is more prominent in aortic valves of female patients with rheumatic heart disease. This sex difference may contribute to the severe valve pathology and worse outcome of female patients.

## Introduction

Rheumatic heart disease (RHD) causes 1.4 million deaths per year in developing countries[1, 2]. The disease starts with group A streptococci infection. Subsequent host autoimmune response to streptococcal antigens crossly reacts to human tissue proteins, affecting the heart, joints, skin, and brain. RHD patients have a high prevalence of severe cardiovascular complications, including rheumatic carditis, atrial fibrillation, pulmonary hypertension, and congestive heart failure. The most serious complication is rheumatic carditis, which often affects the mitral and aortic valve[3–5]. Chronic or recurrent valve inflammation results in valve stenosis and dysfunction, which underlies the disease’s morbidity and mortality. Thus, the essence of RHD is cardiac valve inflammation[1, 5, 6].

Sex differences in the prevalence, symptomatology, risk stratification, efficacy of therapy, and outcome in RHD have been described previously[7]. Especially, nearly two thirds of RHD patients are female[7–10]. RHD patients often develop aortic valve stenosis, regurgitation or both. The end-stage of aortic valve dysfunction eventually requires valve replacement. Despite the importance of aortic valve disease among RHD patients, no study has reported the sex difference in aortic valve pathology. In this study we investigated and compared aortic valve pathology between male and female RHD patients. We related the pathology findings to the clinical characteristics. Our findings suggest augmented inflammation and severe fibrotic aortic valve pathology in female RHD patients.

## Materials and methods

### Study population

Aortic valve samples were collected from RHD patients undergoing aortic valve replacement at The First Affiliated Hospital of Nanjing Medical University from September 2013 to February 2017. The diagnosis of RHD was made by preoperative transthoracic echocardiography and intraoperation diagnosis, and serum test positive for increased anti-streptolysin O (ASO) IgG, rheumatoid factor (RF) and C-reactive protein (CRP). Diseased aortic valves were harvested at the time of the operation and divided into two groups according to sex (19 for male and 20 for female) for further studies. Patients’ medical records including echocardiography were reviewed to assess the cardiovascular risk factors. The study was approved by the Ethics Committee of The First Affiliated Hospital of Nanjing Medical University (Ref. 2015-SRFA-134). All patients signed a written consent before participating the study.

### Serum biomarker measurement

Latex-enhanced turbidimetric immunoassay (Shanghai Ailex Technology Co Ltd) was used for measuring the serum levels of ASO, RF, and CRP. The NT-proBNP level was determined by the fourth-generation Elecsys proBNP assay (Roche Diagnostics). Chemistry modified enzyme method (Shanghai Ailex Technology Co Ltd) was carried out for evaluating the serum levels of total cholesterol (TC), high density lipoproteins-cholesterol (HDL-C), and low density lipoproteins-cholesterol (LDL-C). The serum level of lipoproteins-a [(Lp(a)] was measured using turbidimetric immunoassay (Shanghai Ailex Technology Co Ltd). All assays were carried out using the commercial kits following the manufactures’ instructions, and the optical density of individual immunoreaction products was measured at each specific wavelength by an automatic biochemistry analyzer (Hitachi 7080).

### Histology

Aortic valves freshly isolated during the surgery were fixed in 4% paraformaldehyde (PFA) in phosphate-buffered saline (PBS) overnight at 4°C, dehydrated through an ethanol gradient, treated with xylene, and embedded in paraffin wax. Tissue sections (4 μm) were prepared using a Leica microtome. Hematoxylin and eosin (H&E) staining was used for revealing the basic pathological changes. Masson’s trichrome staining (HT15, Sigma) was performed to evaluate accumulation of collagens and muscle fibers. Von Kossa staining (24633, Polysciences) was performed for revealing calcium deposition using a kit following manufacture’s instruction. Images were taken using the Nikon eclipse 50i microscope. For the morphometric analysis of staining, at least 3 fields of each section were photographed for quantifying the intensity and density of positive signal. We used the Image-Pro Plus 6.0 software to determine the ratio of integrated optical density (IOD) to image area (IOD/AREA). The data were presented as IOD/AREA for the Masson and Von Kossa staining.

### Immunohistochemistry

Transversal 4% PFA-fixed, paraffin-embedded sections (4μm thick) were prepared for subsequent staining. Aortic valve sections were deparaffinized and antigen retrieved with sodium citrate before being incubated with primary antibodies and then secondary antibodies. Rabbit antibodies against human PECAM1 (ab28364, Abcam, 1:50 dilution) for evaluating neovascularization, H2A.X (ab2893, Abcam,1:400) for DNA damage, Ki67 (ab66155, Abcam, 1:200) for cell proliferation, CASP3 (ab2302, Abcam, 1:100) and CASP10 (ab177475, Abcam, 1:100) for apoptotic process. Mouse antibody kits or antibodies against human LCA (kit-0024, Maixin, 1:50), CD3 (kit-0003, Maixin, 1:50), CD4 (ab133616, Abcam, 1:200), CD20 (kit-0001, Maixin, 1:50), CD68 (MAB-0041, Maixin, 1:50), and CD163 (MAB-0206, Maixin, 1:50) were used for lymphocytes, monocytes and macrophages. Rabbit antibodies against the Phospho-p65 at the serine 536 (S536; ab86299, Abcam, 1:200), IFNG (ab175878, Abcam, 1:100) and IL10 (ab34843, Abcam, 1:200) were also used in the study. Affinity-purified secondary anti-rabbit and anti-mouse IgGs were obtained from Vector Laboratories. Immunostained sections were examined using a Nikon eclipse 50i microscope. For statistical analysis of the positive cells stained by each antibody, at least 3 fields of each section were randomly selected, and the intensity and density of positive signal associated with individual cells were determined by using the Image-Pro Plus 6.0 software. The data were presented as IOD/AREA.

### Apoptosis assay

*In situ* end-labeling of DNA fragments (TUNEL) assay kit (11684817910, Roche) was used to determine the apoptotic cells in the diseased valves. The slides were deparaffinized and rehydrated before being incubated with proteinase K solution at 37°C, followed by quenching of endogenous peroxidase by incubation with 3% hydrogen peroxide. Terminal deoxynucleotidyl transferase (TdT) enzyme was then added to label DNA strands, followed by applying anti-digoxigenin peroxidase conjugates to the specimen for color development using peroxidase substrate diaminobenzidine (DAB). Nuclei counterstaining were performed with Harris hematoxylin. Sections were photographed using a Nikon eclipse 50i microscope. For statistical analysis of the TUNEL-positive cells, at least 3 fields of each section were randomly selected. The intensity and density of positive signal associated with cells were determined by using the Image-Pro Plus 6.0 software. The data were presented as IOD/AREA.

### RNA extraction and quantitative real-time PCR

Total RNAs was extracted from aortic valve tissues using the TRIzol solution (217004, Qiagen). cDNA was reverse transcribed from 1μg RNAs using the Superscript II reverse transcriptase Kit (1708891, Bio-Rad). Quantitative reverse transcription real-time PCR (RT-qPCR) was performed on the Applied Biosystems 7500 real-time PCR system using the Power SYBR Green PCR Master Mix (4367659, Life Technology) containing gene specific primers (S1 Table). The fold change of mRNA level was normalized to the level of glyceraldehyde-3-phosphate dehydrogenase (GAPDH) and calculated using the 2^-ΔΔCT^ method. RT-qPCR analysis was performed for 5 or 8 individual samples per group in technical triplicates.

### Statistical analysis

Data were obtained from 5 or 8 patients per group for each experiment and presented as means ± SEM. Statistical analyses were performed with IBM SPSS Statistics Version 2.0 software. Categorical variables were evaluated by the Chi-square test. Gaussian numerical variables were compared with the Student *t* test preceded by Levene’s test for equality of variance. Non-Gaussian numerical variables were assessed by the Mann-Whitney non-parametric test. Two-sided probability (*p*) values < 0.05 were considered statistically significant.

## Results

### High serum level of inflammatory factors and NT-proBNP are associated with RHD Patients

A total of 39 patients with RHD were enrolled in this analysis, including 19 male patients and 20 female patients. The clinical features of the patients were summarized in Table 1. There was no difference in their age and body mass index (BMI) between the two groups. None of female patients had a history of tobacco use, whereas 8 of 19 (42%) male patients were tobacco smokers. Since streptococcal infection is a major pathogenesis of RHD[4, 11], we evaluated the serum level of RF, ASO and CRP between the two groups. The results showed that RHD patients of both groups had higher than normal level of these inflammatory factors (Table 1). The normal level for RF, ASO, and CRP is less than 10.00 IU/mL, 53.80 IU/mL, and 3.19 mg/L, respectively. Of note, female patients showed a significant higher titer of ASO as compared to male patients (73.72 ± 8.58 versus 142.25 ± 38.56 IU/mL, *p* = 0.038). In contrast, both male and female patients had a normal serum level of TC, HDL-C, LDL-C and Lp(a). We also examined the serum level of NT-proBNP, a cardiac function marker, and found that it was markedly elevated in male and female patients, exceeding the normal range in the general population (0–125 ng/L in population younger than 75 year old)[12]. There was, however, no significant difference between the two groups (1037.82 ± 224.70 versus 916.50 ± 215.10, *p* = 0.711). These findings confirm the clinical diagnosis of RHD for the patients enrolled in the study, which was different from the calcific aortic valve stenosis commonly seen in the elderly population and associated with a high serum level of lipids[13]. The higher serum level of ASO in female RHD patients indicates severe autoimmune response in these patients and suggests a possible more prominent inflammation in affecting tissues, such as aortic valve.

**Table 1:** Clinical characteristics of the RHD patients.

Patient characteristics	Male (n = 19)	Female (n = 20)	*p* Value
**Demographic data**			
Age (y)	50.84 ± 2.50	52.70 ± 2.58	0.609
BMI (kg/m^2^)	22.14 ± 0.61	23.61 ± 1.15	0.274
**Aortic valve pathology, No. (%)**			
Pure Stenosis	0 (0.00)	1 (5.00)	1.000
Pure Regurgitation	6 (31.58)	7 (35.00)	0.821
Combined	13 (68.42)	12 (60.00)	0.584
**Mitral valve pathology, No. (%)**			
Pure Stenosis	3 (15.79)	3 (15.00)	1.000
Pure Regurgitation	1 (5.26)	1 (5.00)	1.000
Combined	15 (78.95)	16 (80.00)	1.000
**Comorbidities, No. (%)**			
Atrial Fibrillation	12 (63.16)	10 (50.00)	0.408
Hypertension	4 (21.05)	1 (5.00)	0.308
Coronary artery disease	0 (0.00)	1 (5.00)	1.000
Diabetes mellitus	1 (5.26)	1 (5.00)	1.000
Tobacco use	8 (42.11)	0 (0.00)	0.004 **
**Serum levels**[12, 14] ^#^			
RF (IU/mL)	10.23 ± 0.19	17.48 ± 7.15	0.357
ASO (IU/mL)	73.72 ± 8.58	142.25 ± 38.56	0.038*
CRP (mg/L)	5.02 ± 1.44	5.59 ± 2.24	0.826
NT-proBNP (ng/L)	1037.82 ± 224.70	916.50 ± 215.10	0.711
**Serum lipid levels**[15, 16] ^##^			
TC (mmol/L)	4.09 ± 0.17	4.35 ± 0.21	0.355
HDL-C (mmol/L)	1.11 ± 0.06	1.15 ± 0.05	0.629
LDL-C (mmol/L)	2.58 ± 0.14	2.77 ± 0.20	0.429
Lp(a) (mg/dL)	15.79 ± 3.55	19.63 ± 2.96	0.411

All numeric values are means ± SEM. BMI: body mass index, RF: rheumatoid factor, ASO: anti-streptolysin O antibody, CRP: C-reactive protein, NT-proBNP: N-terminal pro-brain natriuretic peptide, TC: total cholesterol, HDL-C: high density lipoproteins-cholesterol, LDL-C: low density lipoproteins-cholesterol, Lp(a): lipoproteins-α.

*p < 0.05

**p < 0.01.

^#^, References report the normal values of RF, ASO, CRP and NT-proBNP serum levels in Chinese population with similar age to that of patients in the presented study.

^##^, References report the normal values of TC, HDL-C, LDL-C and Lp(a) serum levels in Chinese population with similar age to that of patients in the presented study.

### Left ventricular and aortic valve function in RHD patients

Left ventricular and aortic valve functions in RHD patient was evaluated by echocardiography (Table 2). Consistent with the elevated serum level of NT-proBNP, the results showed a poor left ventricular and aortic valve function among both male and female RHD patients, as indicated by increased left atrial diameter (LAD), left ventricular end-diastolic diameter (LVDd), and aortic valve mean gradient (AVMG). The normal value of these indexes is less than 38 mm, 55 mm (female: 50 mm), and 5 mmHg[17], respectively. In contrast, left ventricular end-systolic diameter (LVDs), left ventricular posterior wall thickness (LVPW), Interventricular septum thickness (IVS), left ventricular fractional shortening (LVFS), and left ventricular ejection fraction (LVEF) were all within the normal range. In addition, RHD patients presented with moderate mitral valve stenosis, elevated pulmonary arterial systolic pressure (PASP), and right atrial diameter (RAD). Interestingly, although female patients had severe autoimmune response, they did not have worse left ventricular and aortic valve function.

**Table 2:** Echocardiographic findings of the RHD patients.

Echocardiographic parameters[17, 18]^#^	Male (n = 19)	Female (n = 20)	*p* Value
Aortic valve mean gradient (AVMG, mmHg)	43.42 ± 7.69	43.85 ± 7.51	0.969
Ascending aorta diameter (Aod, mm)	34.63 ± 1.28	31.80 ± 1.35	0.138
Left atrial diameter (LAD, mm)	51.53 ± 2.19	48.65 ± 1.78	0.311
LV end-diastolic diameter (LVDd, mm)	56.32 ± 1.89	49.70 ± 2.09	0.025 *
LV end-systolic diameter (LVDs, mm)	39.21 ± 1.77	32.60 ± 1.59	0.008 **
LV posterior wall thickness (LVPW, mm)	11.00 ± 0.59	9.50 ± 0.26	0.029 *
Interventricular septum thickness (IVS, mm)	11.63 ± 0.57	9.80 ± 0.41	0.014 *
LV fractional shortening (LVFS, %)	30.70 ± 1.11	34.53 ± 0.80	0.007 **
LV ejection fraction (LVEF, %)	56.94 ± 1.60	63.34 ± 1.12	0.002 **
Right atrial diameter (RAD, mm)	37.67 ± 1.34	35.58 ± 1.57	0.320
RV end-diastolic diameter (RVDd, mm)	34.60 ± 1.47	32.00 ± 1.03	0.181
Mitral valve area (MVA, cm^2^)	1.25 ± 0.12	1.05 ± 0.07	0.159
PA systolic pressure (PASP, mmHg)	49.38 ± 6.26	46.12 ± 3.26	0.642

All numeric values are means ± SEM. LV/RV: left/right ventricle, PA: pulmonary artery.

*p < 0.05

**p < 0.01.

^#^, Two references report the echocardiographic findings in normal population or patients with aortic stenosis.

### Increased collagen expression in aortic valves of female RHD patients

**Fig 1:**
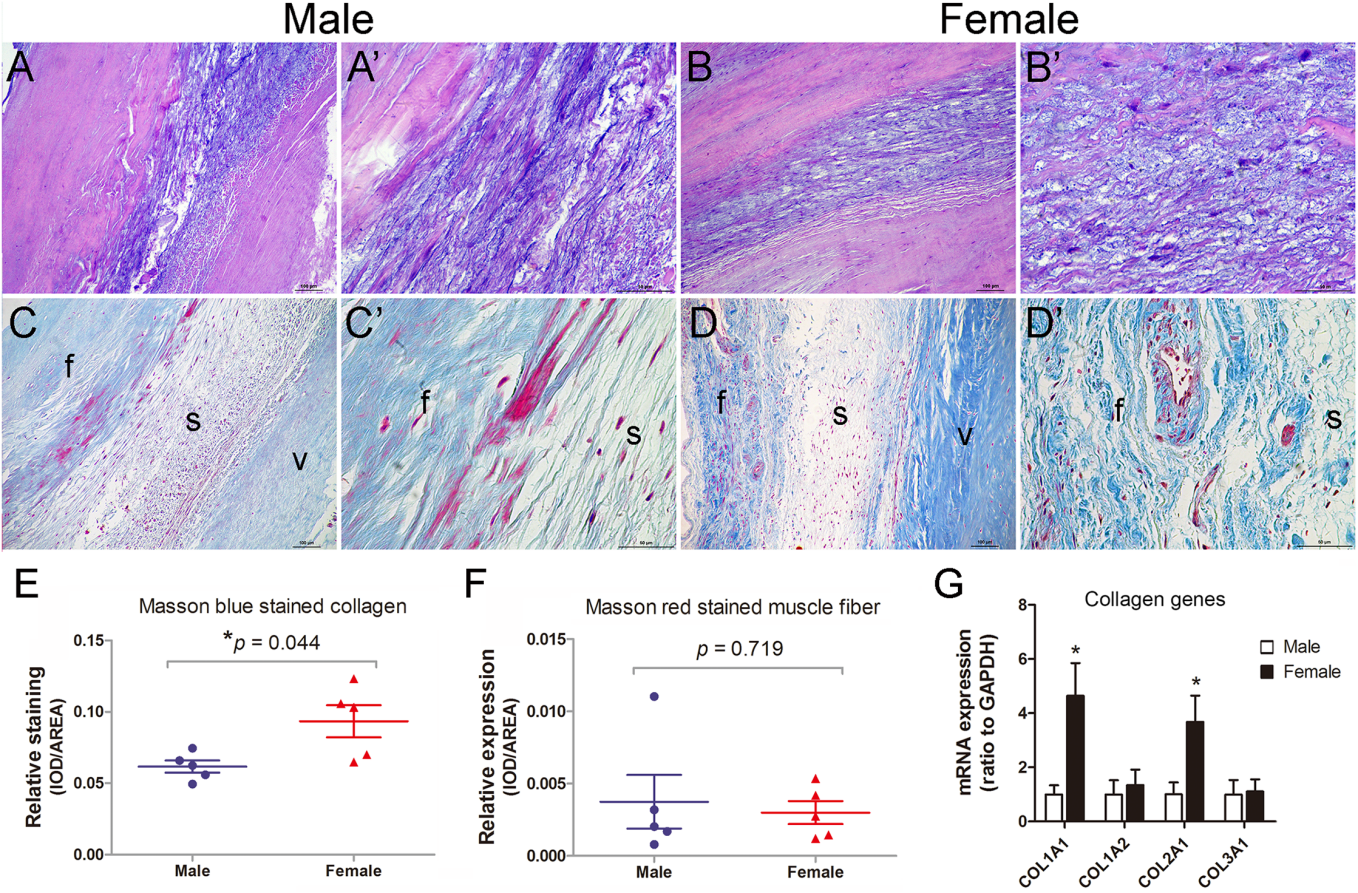
Increased collagen expression in aortic valves of RHD patients. (A-B’): H&E staining shows hyaline degeneration and fibrotic infiltration, consistent with fibromyxomatous degeneration, in aortic valves of male and female RHD patients. (C-F): Masson’s Trichrome staining reveals significantly increased collagen expression (blue staining) in the fibrosa (f) and ventricularis (v) of aortic valves of female patients, while no significant difference in muscle fibers (red staining) is seen between the two groups. (G): RT-qPCR analysis shows increased COL1A1 and COL2A1 expression in aortic valves of female patients. GAPDH expression is used to normalize the expression level. n = 5 patients/group for H&E and Masson staining, n = 8 patients/group for RT-qPCR, *p < 0.05, scale bar = 100μm.

We next examined aortic valve histopathology to determine if there were differences between male and female patients first by H&E and Masson’s Trichrome staining. The results showed that male and female patients were accompanied with hyaline degeneration and infiltration of fractured matrix fibers on H&E staining, suggesting a type of fibromyxomatous degeneration (Fig 1A–1B’). Masson staining revealed increased Masson blue stained collagens within the two outer layers of leaflet, fibrosa and ventricularis, in female patients as compared to male patients (Fig 1C–1D’ and 1E). There was, however, no significant difference in the level of Masson’s red stained muscle fibers (Fig 1C–1D’ and 1F). We also examined the expression of collagen genes by RT-qPCR analysis. The results showed that the mRNA expression of collagen type 1 alpha 1 (COL1A1) and COL2A1 was significantly increased, whereas no change was found for COL1A2 and COL3A1 expression (Fig 1G). These findings indicate sex differences in aortic valve pathology in RHD patients.

### Increased neovascularization in aortic valves of female RHD patients

**Fig 2:**
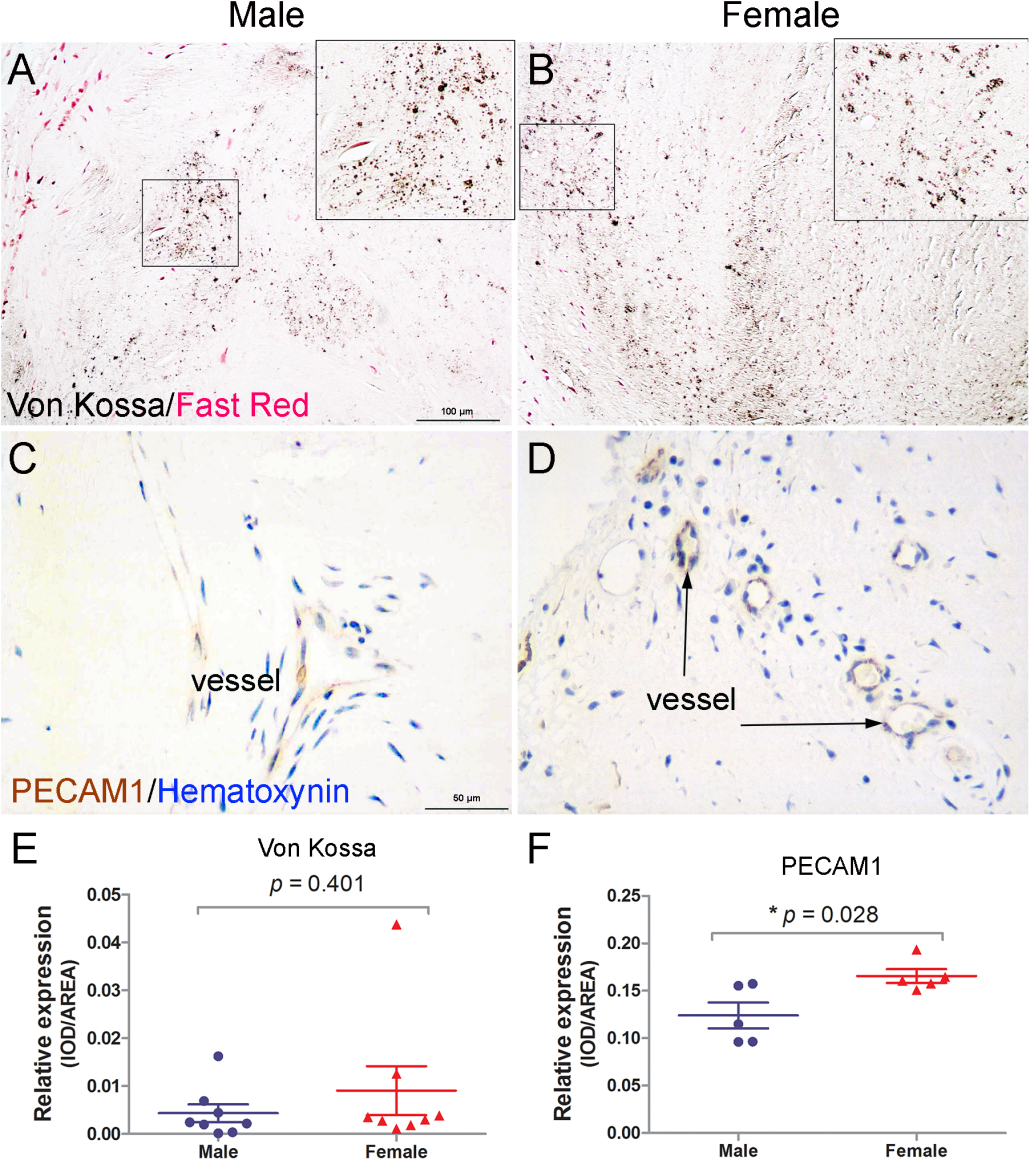
Calcification and neovascularization in aortic valves of RHD patients. (A, B, E): Von Kossa staining (n = 8 patients/group) shows a similar level of granular calcium deposits (asterisk) in aortic valves of male and female patients. (C, D, F): PECAM1 immunostaining (n = 5 patients/group) shows significantly increased neovascularization (arrows) in aortic valves of female patients. *p < 0.05, scale bar = 50μm or 100μm.

Von Kossa and PECAM1 staining were used to determine if there were differences in calcification and neovascularization, respectively, in aortic valves between male and female RHD patients. While the results showed no difference in calcium deposition between the two groups (Fig 2A, 2B and 2E), neovascularization was significantly increased in female patients (Fig 2C, 2D and 2F).

### Different cell fate in aortic valves between male and female RHD patients

**Fig 3:**
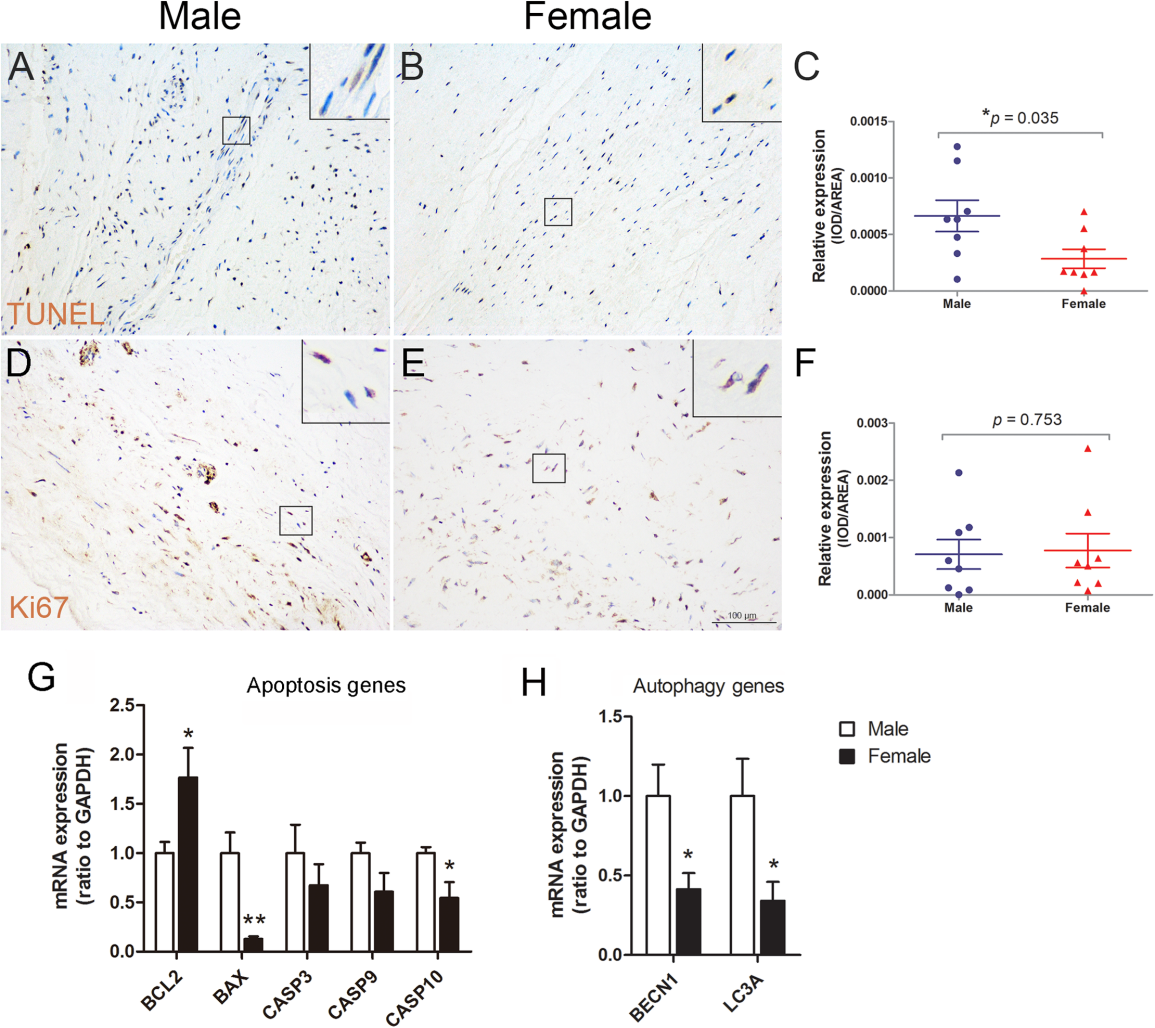
Different cell fate in aortic valves between male and female RHD patients. (A-C): TUNEL assay shows a significant decrease in the intensity and density of apoptotic cells (brown staining) in aortic valves of female patients. (D-F): Ki67 immunostaining indicates no difference in proliferative cells (brown staining) aortic valves between male and female patients. (G): RT-qPCR analysis showing increased BCL2 expression and decreased expression of BAX and CASP10 in aortic valves of female patients. GAPDH expression is used to normalize the expression level. (H): Decreased expression of autophagy genes, BECN1 and LC3A, in aortic valves of female patients. n = 8 patients/group for TUNEL assay and Ki67 staining, n = 5 for RT-qPCR, *p < 0.05, **p < 0.01, scale bar = 100μm.

To better understand the different pathological change in the diseased aortic valves between male and female RHD patients, we examined cell apoptosis using TUNEL assay, CASP3 and CASP10 immunostaining, DNA damage using H2A.X immunostaining, and cell proliferation using Ki67 immunostaining. TUNEL assay showed that apoptosis was significantly decreased in aortic valves of female RHD patients as compared to male patients (Fig 3A–3C), whereas no significant difference was found in the expression level of CASP3, CASP10, and H2A.X in aortic valves between male and female patients (S1 Fig). Ki67 staining also showed no difference in cell proliferation between the two groups (Fig 3D–3F). Further RT-qPCR analysis revealed the mRNA level of B cell lymphoma 2 (BCL2), an anti-apoptotic gene, was significantly upregulated, while the mRNA level of pro-apoptotic genes, BCL2 associated X (BAX) and CASP10, was downregulated in aortic valves of female patients (Fig 3G). Interestingly, the expression of autophagy genes, beclin1 (BECN1) and microtubule associated protein 1 light chain 3 alpha (LC3A), was significantly downregulated in female patients (Fig 3H). These findings suggest that co-downregulation of apoptosis and autophagy in aortic valves of female patients.

### Increased monocyte and macrophage infiltration in aortic valves of female RHD patients

**Fig 4:**
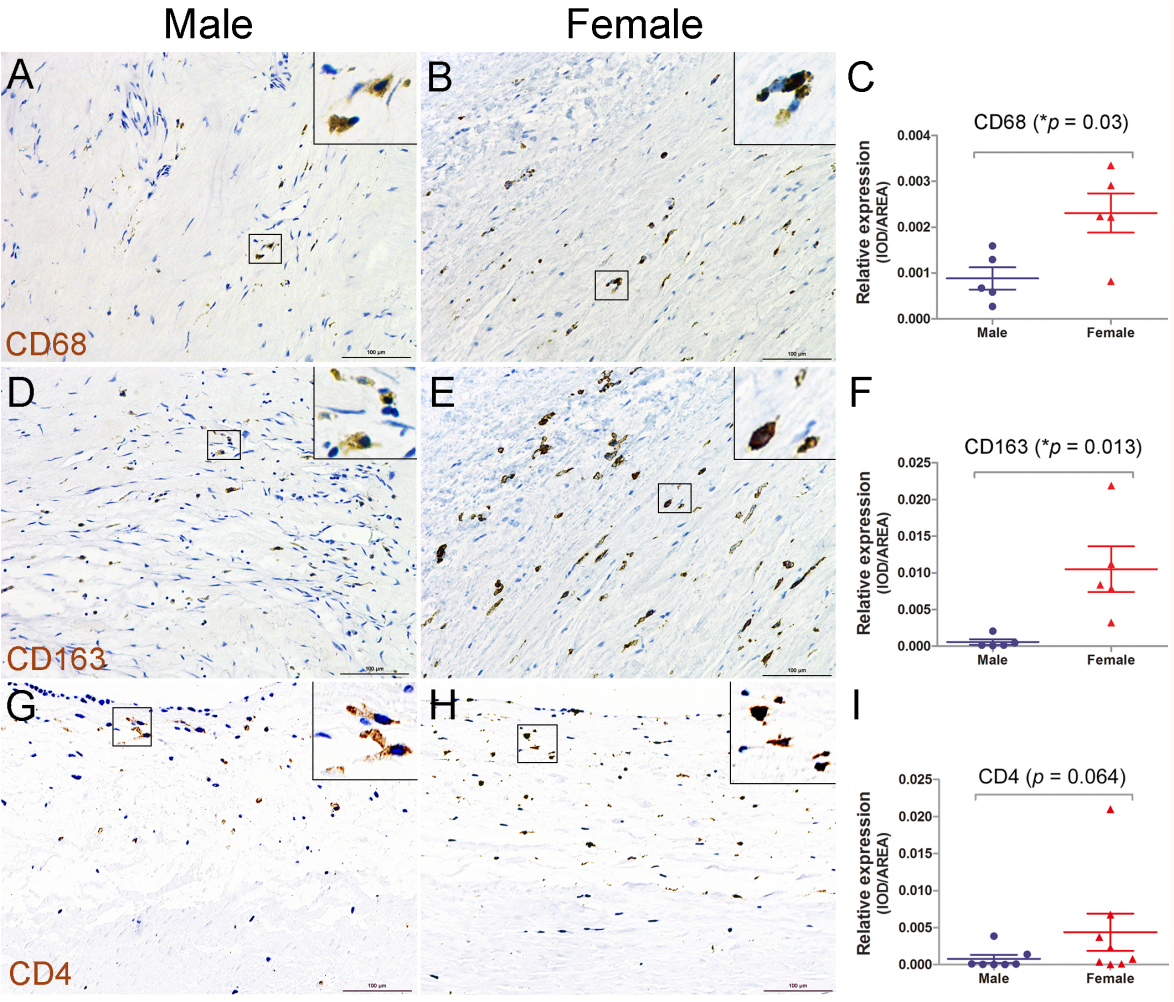
Increased monocyte/macrophage infiltration in aortic valves of female RHD patients. **(**A-C): CD68 staining shows significantly increased infiltration by monocytes/macrophages in aortic valves of female RHD patients as compared to male patients. (D-F): CD163 staining reveals increased monocytes/macrophage infiltration in the valves of female RHD patients. (G-I): CD4 staining shows an increase trend of CD4^+^ T lymphocytes in female patients. n = 5 patients/group for CD68 and CD163 staining, n = 8 for CD4 staining, *p < 0.05, bar = 100μm.

Since a high serum level of inflammatory factors was found in RHD patients, especially in female patients, we then focused on identifying whether infiltration by inflammatory cells was increased in aortic valves of female patients. We examined the leukocytes, T and B lymphocytes, monocytes, and macrophages in the diseased aortic valves of male and female RHD patients by immunostaining. The results showed that there was no significant change in the infiltration of leukocytes stained by LCA antibodies, T lymphocytes stained by CD3 antibodies, and B lymphocytes stained by CD20 antibodies between the two groups (S2 Fig). In contrast, there was significantly increased infiltration of monocytes and macrophages stained by CD68 (Fig 4A–4C) or CD163 antibodies (Fig 4D–4F) in the valves of female patients. In addition, CD4 immunostaining indicated that there was an increase trend in CD4+ T lymphocytes in aortic valves of female patients (Fig 4G–4I). These observations suggest more aggressive inflammatory infiltration of monocytes and macrophages in aortic valves of female patients with RHD.

### Increased inflammatory gene expression in aortic valves of female RHD patients

**Fig 5:**
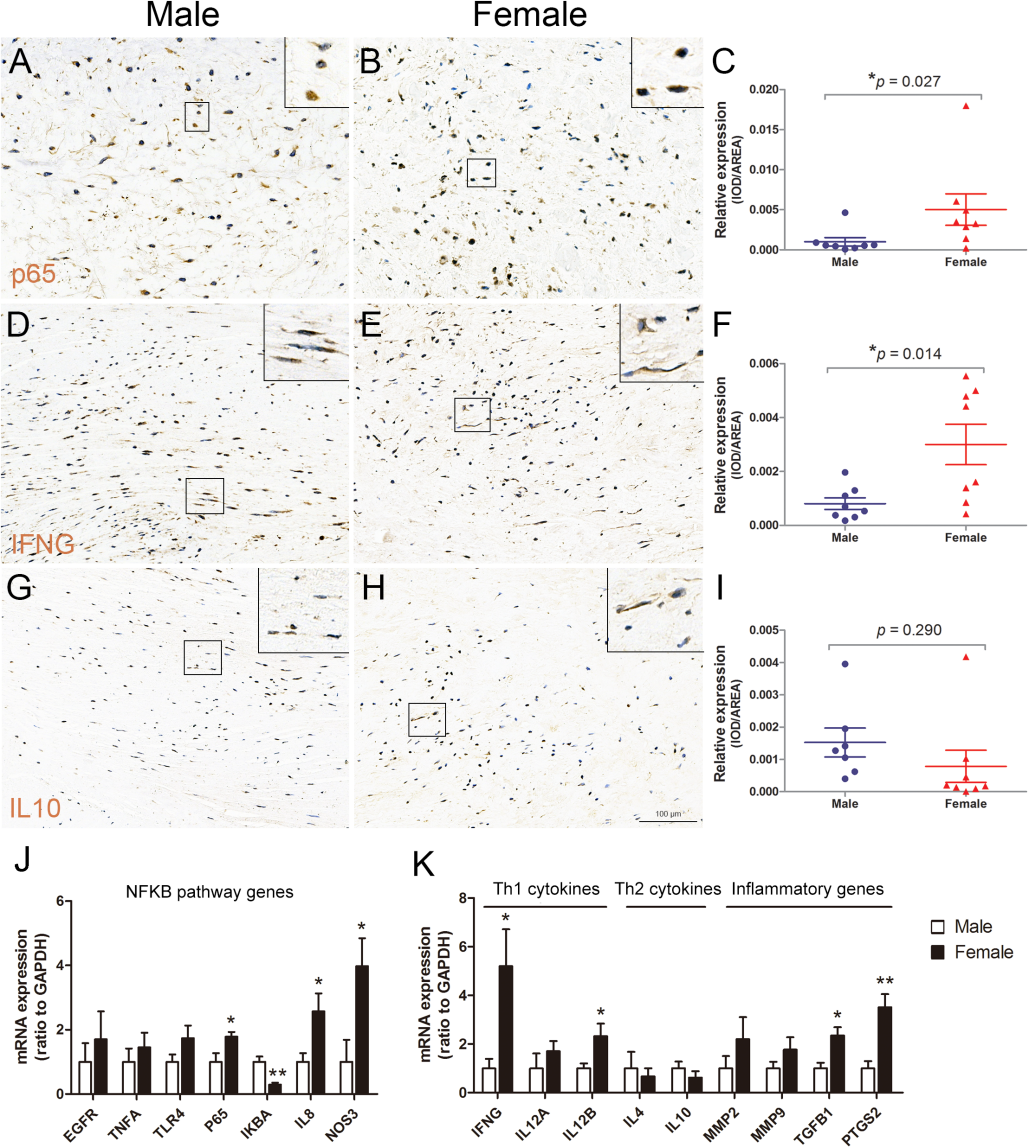
Expression of inflammatory genes in aortic valves of male and female RHD patients. (A-C): Phospho-p65 (S536) immunostaining shows significantly increased NFKB activity in aortic valves of female RHD patients. (D-F): IFNG immunostaining reveals significant upregulation of IFN level in aortic valves of female RHD patients. (G-I): IL10 immunostaining indicates a similar IL10 level in aortic valves between the two groups. (J): RT-qPCR analysis shows increased expression of NFKB pathway genes (p65, IL8, and NOS3) in aortic valves of female patients, whereas the expression of NFKB inhibitor, IKBA, is downregulated. (K): RT-qPCR analysis indicates increased expression of Th1 cytokines (IFNG and IL12B) in aortic valves of female patients, but no difference in the expression of Th2 cytokines between the two groups. The expression of inflammatory related genes (TGFB1 and PTGS2) is also significantly increased in aortic valves of female patients. GAPDH expression is used to normalize the expression level. n = 8 patients/group for all immunostaining, n = 5 or 8 for RT-qPCR, *p < 0.05, **p < 0.01, scale bar = 100μm.

Nuclear factor kappa B (NFKB) plays a central role in the inflammation process[19]. We therefore examined the active phosphorylated NFKB subunit p65 by immunostaining using an antibody against the S536. The result showed that the level of phospo-p65 was increased in aortic valves of female patients when compare with male patients (Fig 5A–5C). The expression level of T help type 1 (Th1) and Th2 cytokines was also determined by immunostaining. The results showed that the protein level of Th1 cytokine, interferon gamma (IFNG), was significantly increased in aortic valves of female patients (Fig 5D–5F), whereas the protein level of Th2 cytokine, interleukin 10 (IL10), was comparable between the two groups (Fig 5G–5I).

We then carried out RT-qPCR analysis to further reveal the mRNA expression of NFKB pathway genes in aortic valves. These genes were epidermal growth factor receptor (EGFR), tumor necrosis factor alpha (TNFA), toll-like receptor 4 (TLR4), NFKB subunit (p65), NFKB inhibitor alpha (IKBA), IL8, and nitric oxide synthase 3 (NOS3). The results showed that the expression of p65, IL8, and NOS3 was significantly elevated in female patients as compared to male patients, while the expression of NFKB inhibitor, IKBA, was decreased in female patients (Fig 5J). Furthermore, we examined the mRNA level of Th1 cytokines (IFNG, IL12A, and IL12B) and Th2 cytokines (IL4 and IL10) and other genes involved in inflammation, including matrix metallopeptidase 2 and 9 (MMP2 and 9), transforming growth factor beta 1 (TGFB1), and prostaglandin-endoperoxide synthase 2 (PTGS2). The results indicated that the expression of IFNG and IL12B was upregulated in aortic valves of female patients, while no change was found for the expression of IL4 and IL10 (Fig 5K). In addition, the expression of TGFB1 and PTGS2 was significantly increased in aortic valves of female patients (Fig 5K). These findings suggest that more pronounced inflammation involved in the NFKB pathway and Th1 cytokines is associated with aortic valve pathology of female patients.

## Discussion

As an autoimmune disease triggered by group A streptococci, RHD presents many cardiovascular manifestations and complications, including cardiac valve deformation and deficiency, infective endocarditis, atrial fibrillation, pulmonary artery hypertension, and heart failure[1, 5]. Aortic valve stenosis and deficiency is a leading factor of morbidity and mortality. Despite its clinical importance, few studies have focused on aortic valve pathology and valve tissue inflammatory response in RHD patients. It is also worth to note that, like coronary artery disease and atrial fibrillation, RHD may have sex differences in etiology, presentation, co-morbidities, management and outcome[20–23]. Previous studies have reported the sex-dependent difference in disease prevalence, risk factor, progression and therapeutic outcome in RHD[7–9]. However, no study has been dedicated to investigate a potential sex difference in aortic valve pathology in RHD.

In this study, we investigated sex differences in aortic valve pathology, immunology and gene expression in RHD patients. We found that inflammatory cells and the expression of inflammatory genes were significantly upregulated in aortic valves in female RHD patients as compared to male patients. NFKB pathway, in particular, was more pronouncedly activated in aortic valves of female RHD patients, suggesting it might play a critical role in augmented autoimmune response in aortic valves. In addition, overexpression of Th1 cytokines may accelerate inflammatory progress in aortic valves of female patients.

Calcific aortic valve disease is known to be the consequence of an active inflammatory process similar to that responsible for atherosclerosis. Both are initiated by basement membrane disruption and lipid deposition, which are mediated by neovascularization and inflammatory factors infiltration[24–27]. It has been reported that the most relevant inflammatory cells in the calcified aortic valves are T lymphocytes and macrophages[28–30]. Aortic valves of RHD are also calcific. Our study demonstrated that the infiltration of monocytes and macrophages was greatly increased in aortic valves of female RHD patients. This finding was consistent with the increased serum level of ASO in female patients.

Beside the increased infiltration of monocytes and macrophages, the augmented expression of inflammatory genes (NFKB, IL8, NOS3, IFNG, IL12B, TGFB1, and PTGS2) was found in aortic valves of female patients. NFKB is activated by proinflammatory cytokines, such as TNFA, IL1B and TLR ligand[31–33]. A link between activation of NFKB and inflammation has been shown in various human diseases[34]. Relevant to this study, early studies have demonstrated that NFKB pathway plays an important role in the development and progression of calcific aortic valve disease[35–38]. Of note, our study is the first to show a potential role of NFKB in RHD induced aortic valve disease. Here we documented the expression of p65, a subunit of NFKB, was significantly upregulated at both transcriptional and posttranscriptional levels in aortic valves of female patients; while the inhibitor of NFKB, IKBA, was downregulated. In support of NFKB over-activation in aortic valves of female RHD patients, the expression of its downstream targets, the IL1B family cytokine, IL8[32], and NOS3 were significantly increased. In contrast, the expression of the upstream regulators of NFKB, EGFR, TNFA and TLR4, was similar between male and female patients. These findings collectively suggest NFKB as a critical inflammatory factor in the pathogenesis of aortic valve stenosis resulting from RHD, especially in female patients.

Th1 and Th2 cells are two main types of CD4^+^ T cells, and cytokines produced by both Th1 and Th2 cells play critical roles in immune homeostasis and inflammatory diseases, which is involved in the activation of NFKB pathway[39, 40]. In this study, we examined Th1 and Th2 cytokines and found that Th1 cytokines IFNG and IL12B were significantly increased in female RHD patients. This finding suggests that Th1 cytokines activate the NFKB pathway likely underlying the worse valve inflammation in female RHD patients.

Apoptosis and proliferation are two necessary morphogenic processes for valve development or disease[41–43]. Although, there was only a decrease trend in the protein level of CASP3 and CASP10 in aortic valves of female patients, TUNEL assay indicated reduced apoptotic cells in aortic valves of female RHD patients. In addition, decreased mRNA level of pro-apoptotic genes (BAX and CASP10) was found in aortic valves of female RHD patients, whereas the mRNA level of anti-apoptotic gene, BCL2, was increased. In addition, we found decreased mRNA expression of autophagy genes (BECN1 and LC3A) in aortic valves of female patients. Future studies on the protein levels of BECN1 and cleaved LC3 are required for determining whether there is sex-dependent difference in the autophagy activity in aortic valves of patients with RHD.

**Fig 6:**
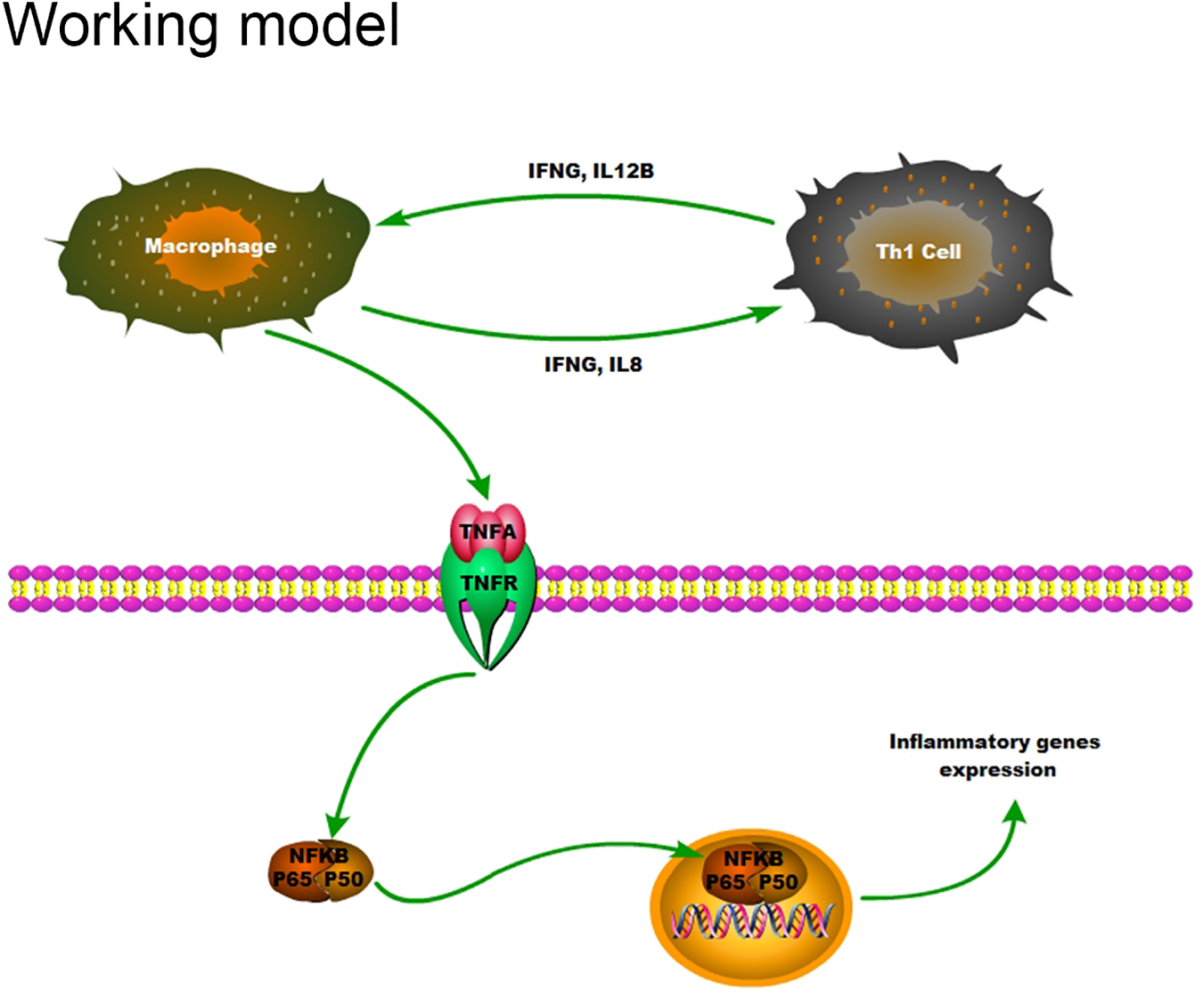
Working model: Upregulation cytokines and NFKB in aortic valve of female RHD patients. Increased monocyte/macrophage infiltration and production of proinflammatory cytokines, especially Th1 cytokines, in aortic valve of female RHD patients activate NFKB. The activated phosphorylated p65 enters nuclei and induces transcription of inflammatory genes, further accelerating the inflammatory process in the diseased aortic valves.

In summary, our findings support that aortic valves of female RHD patients are more inflammatory with increased infiltration of monocytes and macrophages, which produce IFNG and IL8[44]. IFNG and IL12B are also known as major Th1 cytokines, and both are upregulated in aortic valves of female patients. A positive feedback between macrophages and Th1 cells may accelerate proinflammatory cytokines secretion. Together, they activate NFKB through receptors, and the phosphorylation of subunits NFKB p65 then enters nuclei to stimulate transcription of inflammatory genes (Fig 6). Future study will focus on the NFKB pathway as a working model to elucidate whether it play a central role in pathogenesis of aortic valve stenosis in RHD. Such study may provide potential therapeutic targets for the condition.

One limitation of this study is the small sample size. Therefore, we cautiously suggest that the inflammatory and NFKB pathway may contribute to the worse aortic valve pathology seen in female RHD patients. More cases are needed for further characterization of the sex-dependent difference in aortic valve pathology of patients with RHD. In addition, the protein levels of extracellular matrix proteins (COL1A1 and COL2A1) and autophagy markers (BECN1 and LC3) need to be studied by immunohistochemistry and western blot analysis to reveal their potential involvement in aortic valve pathology of RHD patients.

## Supporting information

S1 Fig Immunostaining for apoptotic and DNA damage proteins. (A-C): CASP3 antibody staining shows no significant difference about apoptosis cells in aortic valve of male and female RHD patients. (D-F): CASP10 antibody staining reveals no significant difference about the cell apoptosis (brown staining) between aortic valves of male and female RHD patients. (G-I): H2A.X antibody staining shows no difference in DNA damage in aortic valve cells between the two groups. n = 8 patients/group for CASP3 and CASP8 staining, n = 5 for H2A.X staining, scale bar = 100μm.
(TIF)

S2 Fig Immunostaining for leukocytes and lymphocytes. **(**A-C): Leukocytes stained by LCA antibodies shows no difference between female and male groups. (D-F): T lymphocytes stained by CD3 antibodies reveals no significant difference between the two groups. (G-I): B lymphocytes stained by CD20 antibodies shows no significant difference between female and male patients. n = 5 patients/group, scale bar = 100μm.
(TIF)

S1 Table Primer list for RT-qPCR. (DOCX)
