# Supplementary figures and images for: Sex-dependent aortic valve pathology in patients with rheumatic heart disease
Source: PLoS One. 2017 Jun 29;12(6):e0180230. doi: 10.1371/journal.pone.0180230 (PMC5491156; doi:10.1371/journal.pone.0180230)

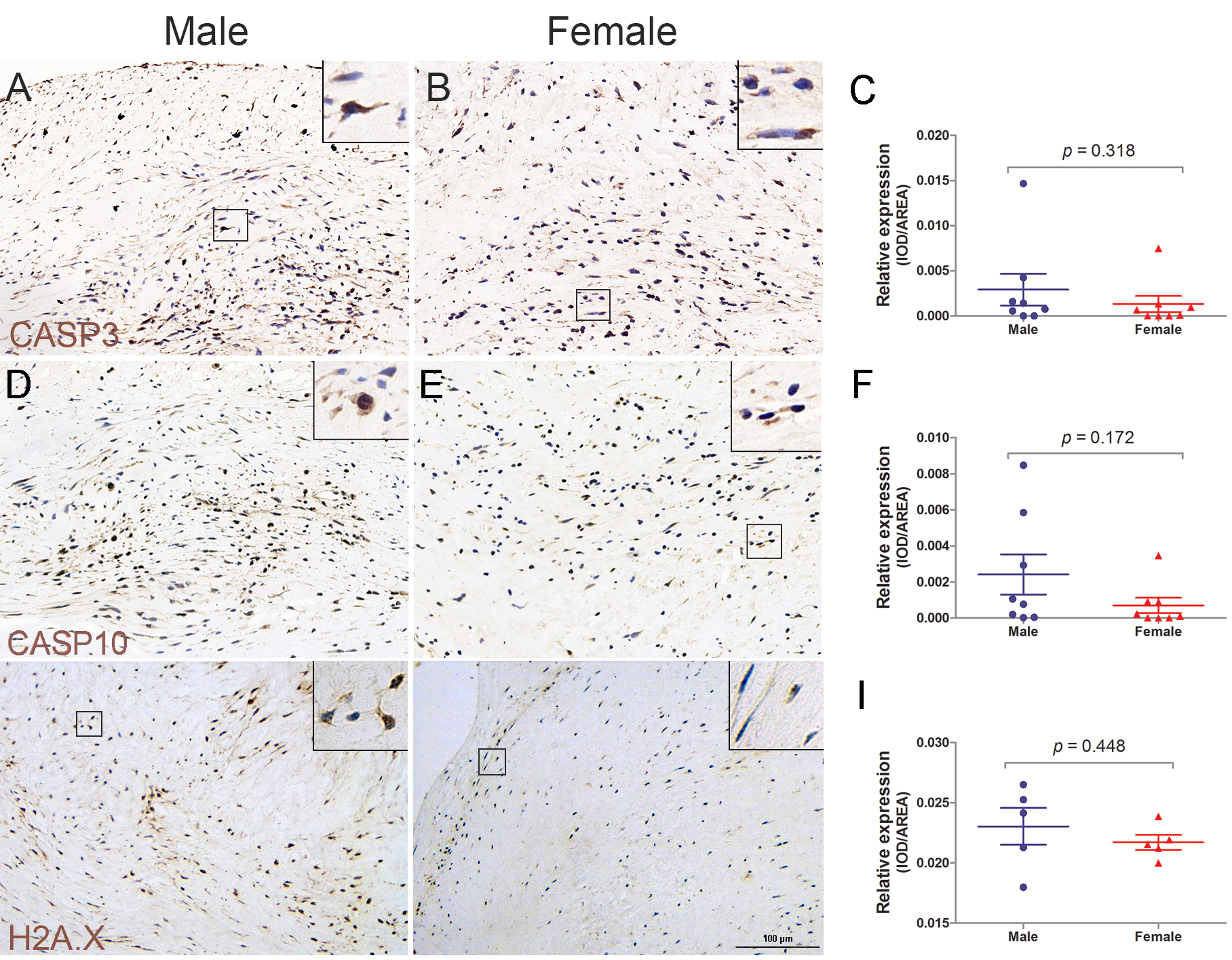

Supplement: S1 Fig — (A-C): CASP3 antibody staining shows no significant difference about apoptosis cells in aortic valve of male and female RHD patients. (D-F): CASP10 antibody staining reveals no significant difference about the cell apoptosis (brown staining) between aortic valves of male and female RHD patients. (G-I): H2A.X antibody staining shows no difference in DNA damage in aortic valve cells between the two groups. n = 8 patients/group for CASP3 and CASP8 staining, n = 5 for H2A.X staining, scale bar = 100μm. (TIF) [file pone.0180230.s001.tif]

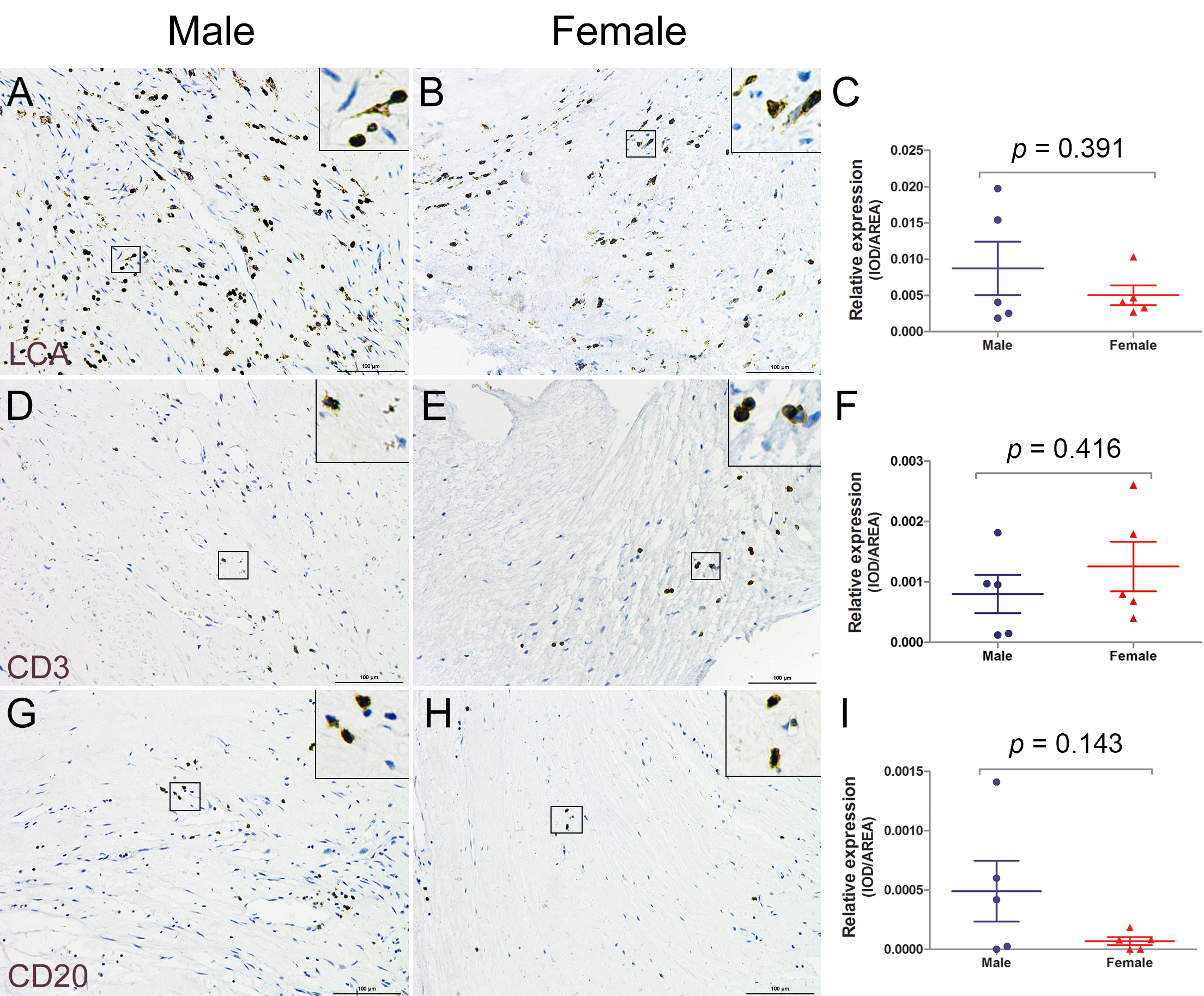

Supplement: S2 Fig — (A-C): Leukocytes stained by LCA antibodies shows no difference between female and male groups. (D-F): T lymphocytes stained by CD3 antibodies reveals no significant difference between the two groups. (G-I): B lymphocytes stained by CD20 antibodies shows no significant difference between female and male patients. n = 5 patients/group, scale bar = 100μm. (TIF) [file pone.0180230.s002.tif]
