# Supplementary material for: Sex-dependent aortic valve pathology in patients with rheumatic heart disease
Source: PLoS One. 2017 Jun 29;12(6):e0180230. doi: 10.1371/journal.pone.0180230 (PMC5491156; doi:10.1371/journal.pone.0180230)
Supplement: S1 Table — (DOCX) [file pone.0180230.s003.docx]

**S1 Table. Primer list for RT-qPCR**

| **Gene name** | **Gene ID** | **Primers (forward/reverse)** |
| --- | --- | --- |
| EGFR | 1956 | AGGCACGAGTAACAAGCTCAC/  ATGAGGACATAACCAGCCACC |
| MMP2 | 4313 | TACAGGATCATTGGCTACACACC/  GGTCACATCGCTCCAGACT |
| MMP9 | 4318 | TGTACCGCTATGGTTACACTCG/  GGCAGGGACAGTTGCTTCT |
| TGFB1 | 7040 | GGCCAGATCCTGTCCAAGC/  GTGGGTTTCCACCATTAGCAC |
| PTGS2 | 5743 | CTGGCGCTCAGCCATACAG/  CGCACTTATACTGGTCAAATCCC |
| NOS3 | 4846 | TGATGGCGAAGCGAGTGAAG/  ACTCATCCATACACAGGACCC |
| p65 | 5970 | AACAGAGAGGATTTCGTTTCCG/  TTTGACCTGAGGGTAAGACTTCT |
| IKBA | 4792 | CTCCGAGACTTTCGAGGAAATAC/  GCCATTGTAGTTGGTAGCCTTCA |
| IL8 | 3576 | TTTTGCCAAGGAGTGCTAAAGA/  AACCCTCTGCACCCAGTTTTC |
| TNFA | 7124 | GAGGCCAAGCCCTGGTATG/  CGGGCCGATTGATCTCAGC |
| TLR4 | 7099 | AGACCTGTCCCTGAACCCTAT/  CGATGGACTTCTAAACCAGCCA |
| BCL2 | 596 | GGTGGGGTCATGTGTGTGG/  CGGTTCAGGTACTCAGTCATCC |
| BAX | 581 | CCCGAGAGGTCTTTTTCCGAG/  CCAGCCCATGATGGTTCTGAT |
| CASP3 | 836 | CATGGAAGCGAATCAATGGACT/  CTGTACCAGACCGAGATGTCA |
| CASP9 | 842 | CTTCGTTTCTGCGAACTAACAGG/  GCACCACTGGGGTAAGGTTT |
| CASP10 | 843 | AGAAACCTGCTCTACGAACTGT/  GGGAAGCGAGTCTTTCAGAAG |
| BECN1 | 8678 | GGTGTCTCTCGCAGATTCATC/  TCAGTCTTCGGCTGAGGTTCT |
| LC3A | 84557 | AACATGAGCGAGTTGGTCAAG/  GCTCGTAGATGTCCGCGAT |
| COL1A1 | 1277 | GAGGGCCAAGACGAAGACATC/  CAGATCACGTCATCGCACAAC |
| COL1A2 | 1278 | GTTGCTGCTTGCAGTAACCTT/  AGGGCCAAGTCCAACTCCTT |
| COL2A1 | 1280 | TGGACGATCAGGCGAAACC/  GCTGCGGATGCTCTCAATCT |
| COL3A1 | 1281 | GGAGCTGGCTACTTCTCGC/  GGGAACATCCTCCTTCAACAG |
| IFNG | 3458 | TCGGTAACTGACTTGAATGTCCA/  TCGCTTCCCTGTTTTAGCTGC |
| IL12A | 3592 | CCTTGCACTTCTGAAGAGATTGA/  ACAGGGCCATCATAAAAGAGGT |
| IL12B | 3593 | ACCCTGACCATCCAAGTCAAA/  TTGGCCTCGCATCTTAGAAAG |
| IL4 | 3565 | CCAACTGCTTCCCCCTCTG/  TCTGTTACGGTCAACTCGGTG |
| IL10 | 3586 | GACTTTAAGGGTTACCTGGGTTG/  TCACATGCGCCTTGATGTCTG |
